# Supplementary material for: Differences in depression prevalence among older adults in China before and during the COVID-19 pandemic: a systematic review and meta-analysis
Source: PeerJ. 2025 Apr 11;13:e19251. doi: 10.7717/peerj.19251 (PMC11995894; doi:10.7717/peerj.19251)
Supplement: Supplemental Information 2 [file peerj-13-19251-s002.docx]

**Supplementary Material**

**Method S1 Relative risk (RR) of depression (2017–2019, 2020.1–4, 2020.5–12, 2021–2023)**

Using the prevalence of depression from 2017–2019 as a reference, the relative risk (RR) for 2017–2019 is set as 1. The RRs for other periods are calculated relative to this baseline:

a. RR for January to April 2020:

$$RR_{2020.1-4}=\frac{P_{2020.1-4}}{P_{2017-2019}}$$

Here, *P*_2017–2019_ represents the prevalence of depression from 2017–2019, *P*_2020.1–4_ represents the prevalence of depression from January to April 2020, and *P*_2020.5–12_ represents the prevalence of depression from May to December 2020.

b. RR for May to December 2020:

$$RR_{2020.5-12}=\frac{P_{2020.5-12}}{P_{2017-2019}}$$

Here, *P*_2020.5–12_ represents the prevalence of depression from May to December 2020.

c. RR for 2021 to 2023:

$$RR_{2021-2023}=\frac{P_{2021-2023}}{P_{2017-2019}}$$

Here, *P_2_*_021–2023_ represents the prevalence of depression from 2021–2023.

Using the prevalence of depression from January to April 2020 as a reference, the relative risk (RR) for this period is set as 1. The RRs for other periods are calculated relative to this baseline:

a. RR for May to December 2020:

$$RR_{2020.5-12}=\frac{P_{2020.5-12}}{P_{2020.1-4}}$$

b. RR for 2021 to 2023:

$$RR_{2021-2023}=\frac{P_{2021-2023}}{P_{2020.1-4}}$$

**Figure S1 Analytical framework**

**
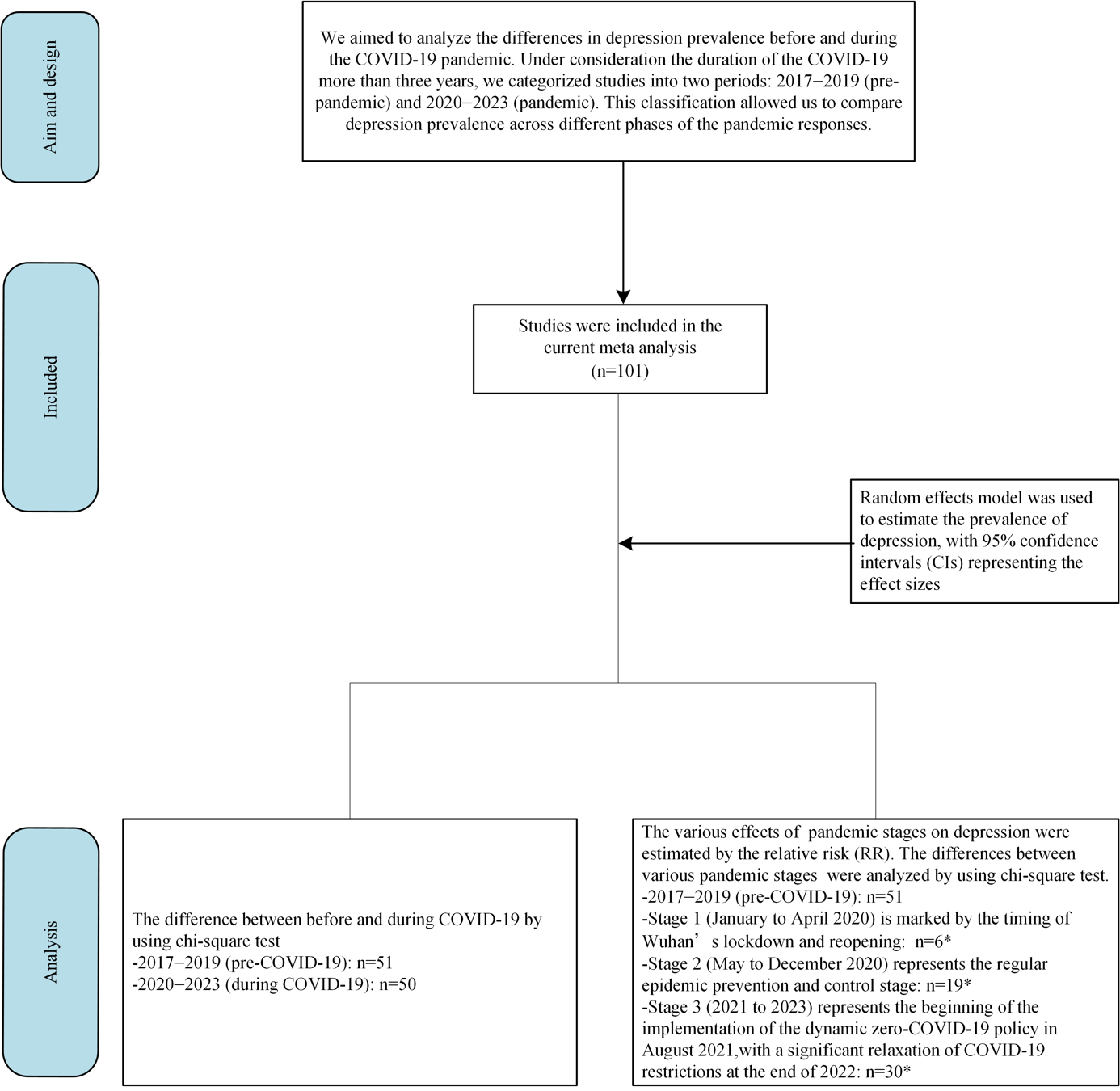
**

* If the survey period of a study spanned from January 2020 to September 2020, its results were included in both Stage 1 and Stage 2. Similarly, results from studies conducted between October 2020 and December 2021 were incorporated into both Stage 2 and Stage 3.

**Table S1 Quality of assessment**

| **Item** | **Yes** | **No** | **Unclear** |
| --- | --- | --- | --- |
| (1) Define the source of information (survey, record review). | 101 | 0 | 0 |
| (2) List inclusion and exclusion criteria for exposed and unexposed subjects (cases and controls) or refer to previous publications. | 101 | 0 | 0 |
| (3) Indicate time period used for identifying patients. | 100 | 1 | 0 |
| (4) Indicate whether or not subjects were consecutive if not population based. | 98 | 3 | 0 |
| (5) Indicate if evaluators of subjective components of the study were masked to other aspects of the status of the participants. | 0 | 101 | 0 |
| (6) Describe any assessments undertaken for quality assurance purposes (e.g., test/retest of primary outcome measurements). | 94 | 6 | 1 |
| (7) Explain any patient exclusions from the analysis. | 68 | 33 | 0 |
| (8) Describe how confounding was assessed and/or controlled. | 90 | 4 | 7 |
| (9) If applicable, explain how missing data were handled in the analysis. | 33 | 68 | 0 |
| (10) Summarize patient response rates and completeness of data collection. | 101 | 0 | 0 |
| (11) Clarify what follow-up, if any, was expected and the percentage of patients for which incomplete data or follow-up was obtained. | 0 | 101 | 0 |

**Table S2 Characteristics of the 101 studies included in the current study**

| **No.** | **Study** | **Language*** | **Location** | **Survey Year**** | **Age** | **Diagnostic Tool***** | **Sample**  **Size/Cases** | **Rate**  **(%)** | **Female**  **(N, %)** | **Rural**  **(N, %)** | **Score of Quality** |
| --- | --- | --- | --- | --- | --- | --- | --- | --- | --- | --- | --- |
| 1 | Ding et al., 2018 | CH | Anhui | 2017 | ≥65 | SDS-20 | 1491/315 | 21.13 | 730(48.96) | NR | 8 |
| 2 | Chen et al., 2019 | CH | Henan | 2017 | ≥60 | GDS | 488/116 | 23.77 | 302(61.89) | 258(52.87) | 9 |
| 3 | Yang et al., 2021 | CH | Yunnan | 2017 | ≥60 | GDS-30 | 1629/195 | 11.97 | 941(57.77) | 1629(100) | 8 |
| 4 | Liu et al., 2022 | CH | Hainan | 2017-2018 | ≥60 | GDS-15 | 2620/99 | 3.78 | 1610(61.45) | NR | 9 |
| 5 | Wu et al., 2020 | EN | Henan | 2017 | ≥60 | PHQ-2 | 3759/150 | 3.99 | NR | 3759(100) | 10 |
| 6 | Chu et al., 2020 | EN | Jiangsu | 2017 | ≥70 | GDS-15 | 1264/133 | 10.52 | 666(52.69) | NR | 9 |
| 7 | Hu et al., 2022 | EN | Zhejiang | 2017-2018 | ≥60 | SDS | 364/225 | 61.81 | 184(50.55) | 364(100) | 9 |
| 8 | Jiang et al., 2022 | EN | Beijing,  Tianjin,  Hebei,  Jiangsu,  Zhejiang,  Shanghai,  Guangdong | 2017-2018 | ≥65 | PHQ-9 | 1851/158 | 8.54 | NR | NR | 9 |
| 9 | Gu et al., 2020 | EN | Jiangsu | 2017-2018 | ≥60 | GDS-15 | 172/32 | 18.60 | 110(63.95) | NR | 9 |
| 10 | Yuan et al., 2024 | EN | National | 2017-2018 | ≥65 | CESD-10 | 11245/1319 | 11.73 | 5966(53.05) | 7907(70.32) | 10 |
| 11 | Xiong et al., 2022 | EN | National | 2017-2018 | ≥80 | CESD-10 | 1154/277 | 24.00 | 627(54.33) | 846(73.31) | 10 |
| 12 | Rong et al., 2021 | EN | Anhui | 2018 | ≥60 | GDS-30 | 3336/1766 | 52.94 | 1696(50.84) | 3336(100) | 9 |
| 13 | Sun et al., 2020 | CH | Hebei | 2018 | ≥60 | GDS-15 | 4889/3445 | 70.46 | 2600(53.18) | 4889(100) | 9 |
| 14 | Dai et al., 2019 | EN | Zhejiang | 2018 | ≥60 | GDS-15 | 3757/974 | 25.92 | 1887(50.23) | 2313(61.57) | 10 |
| 15 | Zhang et al., 2021 | CH | Hebei,  Zhejiang,  Shaanxi,  Hunan | 2018-2019 | ≥65 | GDS-30 | 7072/639 | 9.04 | NR | NR | 9 |
| 16 | Yan et al., 2022 | EN | Tianjin | 2018-2019 | ≥60 | SDS | 3304/384 | 11.62 | 1818(55.02) | 3304(100) | 10 |
| 17 | Lin et al., 2021 | EN | Tianjin | 2018-2019 | ≥60 | SDS-20 | 4933/602 | 12.20 | 2681(54.35) | 4933(100) | 9 |
| 18 | Zhai et al., 2023 | CH | National | 2018-2019 | ≥60 | CESD-8 | 6302/537 | 8.52 | 3089(49.02) | 4387(69.61) | 8 |
| 19 | Liu et al., 2021 | EN | Shandong | 2018 | ≥60 | GDS-5 | 936/97 | 10.36 | 646(69.02) | NR | 9 |
| 20 | Lin et al., 2020 | EN | Guangdong | 2018 | ≥60 | SDS-20 | 335/92 | 27.46 | 221(65.97) | 335(100) | 7 |
| 21 | Li et al., 2022 | EN | National | 2018 | ≥60 | CESD-10 | 8104/3101 | 38.27 | 3944(48.67) | 5974(73.72) | 10 |
| 22 | Wang et al., 2022 | EN | Hebei | 2018 | ≥65 | GDS-30 | 1882/105 | 5.58 | NR | NR | 10 |
| 23 | Gao et al., 2022 | EN | National | 2018 | ≥60 | CESD-10 | 5934/2728 | 45.97 | NR | NR | 9 |
| 24 | Zhao et al., 2020 | EN | National | 2018 | ≥60 | GDS-15 | 4103/803 | 19.57 | 2392(58.30) | NR | 10 |
| 25 | Peng et al., 2022 | EN | Shanghai | 2018 | ≥60 | GDS-30 | 1066/722 | 67.73 | 627(58.82) | NR | 8 |
| 26 | Ma et al., 2020 | CH | Sichuan | 2018 | ≥60 | CESD-10 | 934/245 | 26.23 | NR | NR | 9 |
| 27 | Zhang et al., 2022 | EN | Shanxi | 2018 | ≥60 | GDS-15 | 3637/1213 | 33.35 | 1773(48.75) | NR | 8 |
| 28 | Li et al., 2023 | EN | National | 2018 | ≥60 | CESD-9 | 11418/5827 | 51.03 | 5682(49.76) | NR | 9 |
| 29 | Li et al., 2021 | CH | Hebei | 2018-2019 | ≥60 | GDS-15 | 582/224 | 38.49 | 322(55.33) | NR | 9 |
| 30 | You et al., 2023 | EN | Hunan | 2018-2019 | ≥60 | GDS-15 | 234/103 | 44.02 | 234(100) | 234(100) | 9 |
| 31 | Jiang et al., 2022 | EN | Shandong | 2019 | ≥60 | GDS-15 | 3769/412 | 10.93 | 2375(63.01) | NR | 8 |
| 32 | Ding et al., 2022 | EN | Shanghai | 2019 | ≥60 | SDS-20 | 1429/352 | 24.63 | 1429(100) | NR | 10 |
| 33 | Qiu et al., 2020 | EN | Jiangsu | 2019 | ≥60 | PHQ-9 | 5090/769 | 15.11 | 2449(48.11) | 5090(100) | 9 |
| 34 | Li et al., 2022 | CH | Shanghai | 2019 | ≥60 | GDS-30 | 2518/337 | 13.38 | 1405(55.80) | NR | 9 |
| 35 | Hou et al., 2022 | EN | Anhui | 2019 | ≥60 | PHQ-9 | 5822/2279 | 39.14 | 3169(54.43) | NR | 9 |
| 36 | Yang et al., 2021 | EN | Anhui | 2019 | ≥60 | CESD-20 | 871/296 | 33.98 | 435(49.94) | 871(100) | 9 |
| 37 | Liang et al., 2021 | EN | Shanghai | 2019 | ≥60 | GDS-30 | 2525/339 | 13.43 | 1410(55.84) | NR | 9 |
| 38 | Wang et al., 2021 | CH | Liaoning | 2019 | ≥60 | GDS-30 | 424/212 | 50.00 | 296(69.81) | NR | 9 |
| 39 | Li et al., 2021 | CH | Shanghai | 2019 | ≥60 | PHQ-9 | 1521/676 | 44.44 | 834(54.83) | NR | 9 |
| 40 | Zhao et al., 2022 | EN | Liaoning | 2019 | ≥60 | GDS-30 | 522/299 | 57.28 | 364(69.73) | NR | 8 |
| 41 | Xu et al., 2022 | EN | Guangdong | 2019 | ≥60 | PHQ-9 | 391/17 | 4.35 | 156(39.90) | NR | 8 |
| 42 | Han et al., 2021 | CH | Shandong | 2019 | ≥60 | GDS-15 | 915/155 | 16.94 | 387(42.30) | NR | 9 |
| 43 | Zhang et al., 2021 | CH | Chongqing | 2019 | ≥60 | GDS-30 | 290/88 | 30.34 | 146(50.34) | 0 | 9 |
| 44 | Wang et al., 2022 | CH | Liaoning,  Guangdong, Henan | 2019 | ≥60 | PHQ-9 | 14335/2215 | 15.45 | 8212(57.29) | 7879(54.96) | 9 |
| 45 | Liu et al., 2021 | CH | Hunan | 2019 | ≥65 | PHQ-9 | 13362/1174 | 8.79 | 7355(55.04) | 3404(25.48) | 9 |
| 46 | Xiong et al., 2023 | CH | Guizhou | 2019 | ≥60 | PHQ-2 | 1615/632 | 39.13 | NR | NR | 9 |
| 47 | Chang et al., 2023 | CH | Anhui | 2019 | ≥60 | PHQ-9 | 6083/1892 | 31.10 | 3318(54.55) | 3067(50.42) | 8 |
| 48 | Zeng et al., 2023 | CH | Guizhou | 2019 | ≥60 | PHQ-2 | 1654/109 | 6.59 | 961(58.10) | NR | 10 |
| 49 | Wang et al., 2024 | EN | Anhui | 2019 | ≥60 | PHQ-9 | 6211/1900 | 30.59 | NR | NR | 8 |
| 50 | Wang et al., 2023 | EN | Tianjin | 2018-2019 | ≥60 | GDS-15 | 1180/140 | 11.86 | 647(54.83) | NR | 8 |
| 51 | Guo et al., 2022 | CH | Jiangsu | 2019-2020 | ≥60 | CESD-10 | 2345/554 | 23.62 | 1168(49.81) | 1541(65.71) | 9 |
| 52 | Lu et al., 2022 | CH | Guangdong | 2020 | ≥65 | GDI | 2102/779 | 37.06 | 1024(48.72) | NR | 9 |
| 53 | Wang et al., 2020 | EN | National | 2020 | ≥65 | PHQ-9 | 3560/378 | 10.62 | NR | NR | 8 |
| 54 | Wu et al., 2020 | EN | National | 2020 | ≥60 | HADS | 6011/2838 | 47.21 | NR | NR | 8 |
| 55 | Zhou et al., 2021 | EN | National | 2020 | ≥60 | PQEEPH | 1278/177 | 13.85 | 707(55.32) | 690(53.99) | 10 |
| 56 | Wang et al., 2021 | EN | National | 2020 | ≥65 | PHQ-9 | 5701/853 | 14.96 | NR | NR | 8 |
| 57 | Wang et al., 2020 | EN | National | 2020 | ≥60 | SRD | 852/65 | 7.63 | NR | NR | 8 |
| 58 | Liu et al., 2022 | EN | National | 2020 | ≥60 | PHQ-9 | 1753/90 | 5.13 | NR | NR | 8 |
| 59 | Li et al., 2022 | EN | Jiangsu | 2020 | ≥60 | GDS-15 | 288/37 | 12.85 | 152(52.78) | NR | 9 |
| 60 | Liang et al., 2021 | EN | Hubei | 2020 | ≥60 | CESD-10 | 516/159 | 30.81 | 299(57.95) | NR | 9 |
| 61 | Hou et al., 2022 | EN | Jiangsu | 2020 | ≥60 | GDS-5 | 527/52 | 9.87 | 266(50.47) | NR | 9 |
| 62 | Mao et al., 2022 | EN | Shanghai | 2020 | ≥60 | CESD-10 | 472/356 | 75.42 | 266(56.36) | NR | 8 |
| 63 | Qin et al., 2022 | CH | Shandong | 2020 | ≥65 | PHQ-9 | 1004/93 | 9.26 | 561(55.88) | 1004(100) | 9 |
| 64 | Liu et al., 2022 | CH | Hunan | 2020-2021 | ≥60 | CESD-10 | 267/113 | 42.32 | 143(53.56) | 267(100) | 9 |
| 65 | Liu et al., 2021 | EN | Hubei | 2020 | ≥65 | GDS-30 | 470/66 | 14.04 | 273(58.09) | NR | 9 |
| 66 | Yu et al., 2024 | CH | Shanghai | 2020 | ≥60 | GDS | 3767/50 | 1.33 | 2265(60.13) | NR | 9 |
| 67 | Zhang et al., 2024 | CH | National | 2020 | ≥60 | CESD-10 | 8240/3373 | 40.93 | 4074(49.44) | NR | 8 |
| 68 | Liu et al., 2022 | CH | Shanghai | 2020-2021 | ≥80 | GDS-5 | 411/165 | 40.15 | 218(53.04) | NR | 9 |
| 69 | Qin et al., 2022 | CH | Guangxi | 2020-2021 | ≥60 | GDS-15 | 1946/316 | 16.24 | 967(49.69) | 692(35.56) | 9 |
| 70 | Ding et al., 2022 | EN | Guangdong | 2020-2021 | ≥60 | PHQ-9 | 506/84 | 16.60 | 310(61.26) | NR | 8 |
| 71 | Cui et al., 2022 | CH | Xinjiang | 2020-2021 | ≥60 | GDS-15 | 315/55 | 17.46 | NR | NR | 8 |
| 72 | Zhao et al., 2023 | CH | Hebei | 2020-2021 | ≥75 | GDS | 537/185 | 34.45 | 249(46.37) | NR | 7 |
| 73 | Hao et al., 2023 | EN | National | 2021 | ≥60 | PHQ-9 | 1122/529 | 47.15 | 553(49.29) | NR | 9 |
| 74 | Zong et al., 2022 | CH | Shandong | 2021 | ≥60 | PHQ-9 | 3192/198 | 6.20 | 1981(62.06) | NR | 10 |
| 75 | Xu, 2022 | CH | Henan | 2021 | ≥60 | SDS-20 | 235/65 | 27.66 | 157(66.81) | NR | 9 |
| 76 | Lu et al., 2023 | CH | Hunan | 2021 | ≥65 | PHQ-9 | 1173/438 | 37.34 | 629(53.62) | NR | 9 |
| 77 | Yu et al., 2022 | CH | Ningxia | 2021 | ≥65 | PHQ-9 | 1937/760 | 39.24 | 1018(52.56) | 1045(53.95) | 9 |
| 78 | Li et al., 2022 | EN | National | 2021 | ≥60 | PHQ-9 | 1147/551 | 48.04 | 566(49.35) | NR | 8 |
| 79 | Li et al., 2022 | EN | Jiangsu | 2021 | ≥60 | PHQ-9 | 383/39 | 10.18 | NR | NR | 7 |
| 80 | Li et al., 2022 | EN | Shandong | 2021 | ≥60 | DASS-21 | 613/42 | 6.85 | 448(73.08) | 525(85.64) | 10 |
| 81 | Li et al., 2021 | CH | Yunnan | 2021 | ≥60 | GDS | 1409/733 | 52.02 | 719(51.03) | NR | 8 |
| 82 | He et al., 2022 | EN | Sichuan | 2022 | ≥60 | GDS-15 | 2026/609 | 30.06 | NR | NR | 9 |
| 83 | Chen et al., 2023 | CH | Yunnan | 2022 | ≥60 | GDS-30 | 1418/119 | 8.39 | 749(52.82) | 1418(100) | 8 |
| 84 | Zhao et al., 2024 | CH | Beijing | 2022 | ≥65 | GDS | 3575/573 | 16.03 | 1793(50.15) | NR | 10 |
| 85 | Liu et al., 2024 | CH | Sichuan | 2022 | ≥60 | GDS | 606/85 | 14.03 | 332(54.79) | NR | 9 |
| 86 | Liu et al., 2023 | CH | Xinjiang | 2022 | ≥60 | GDS-15 | 395/30 | 7.60 | 254(64.30) | NR | 10 |
| 87 | Yang et al., 2024 | CH | Shanxi | 2023 | ≥60 | CESD-10 | 1851/915 | 49.43 | 905(48.89) | NR | 8 |
| 88 | Liu et al., 2024 | CH | Xizang | 2022 | ≥65 | HAMD | 527/122 | 23.15 | 247(46.87) | NR | 8 |
| 89 | Huang et al., 2024 | CH | Hunan | 2023 | ≥60 | CESD-10 | 6004/2048 | 34.11 | 3090(51.47) | 3156(52.56) | 10 |
| 90 | Lin et al., 2023 | CH | Shanxi | 2021-2022 | ≥60 | GDS-15 | 665/72 | 10.83 | 384(57.74) | NR | 8 |
| 91 | Su et al., 2023 | CH | Anhui | 2022-2023 | ≥65 | GDS-15 | 388/70 | 18.04 | NR | NR | 8 |
| 92 | Yang et al., 2023 | CH | Guangdong | 2020 | ≥65 | GDI | 2112/626 | 29.64 | 1080(51.14) | NR | 8 |
| 93 | Ge et al., 2023 | CH | National | 2020 | ≥60 | CESD | 4361/1282 | 29.40 | 2075(47.58) | 2170(49.76) | 8 |
| 94 | Jiang et al., 2024 | EN | Anhui | 2021-2022 | ≥60 | GDS-15 | 648/130 | 20.06 | NR | NR | 10 |
| 95 | Zhang et al., 2024 | EN | Jilin | 2022 | ≥65 | PHQ-9 | 1866/255 | 13.67 | 1247(73.87) | NR | 8 |
| 96 | Hu et al., 2023 | EN | Guangdong | 2020-2021 | ≥60 | PHQ-9 | 471/65 | 13.80 | 284(60.30) | NR | 7 |
| 97 | Gan et al., 2024 | EN | Hubei, Guangdong | 2020-2021 | ≥60 | GDS | 317/50 | 15.77 | 243(76.66) | NR | 9 |
| 98 | Xiong et al., 2024 | EN | National | 2021 | ≥60 | PHQ-9 | 165/34 | 20.61 | NR | NR | 8 |
| 99 | Guo et al., 2024 | EN | Hebei | 2022 | ≥60 | HADS | 195/100 | 51.28 | 119(61.03) | NR | 8 |
| 100 | Peng et al., 2024 | EN | Guangdong | 2020-2021 | ≥65 | PHQ-9 | 5331/554 | 10.39 | 2859(53.63) | NR | 9 |
| 101 | He et al., 2024 | EN | Guangdong | 2022 | ≥60 | PHQ-9 | 8436/212 | 2.51 | NR | NR | 10 |

* Language: CH=Chinese, EN=English.

** Survey year is the year the data were collected.

*** Diagnostic Tool: SDS: *Self-rating Depression Scale*; GDS: *Geriatric Depression Scale*; PHQ: *Patient Health Questionnaire*; CESD: *Center for Epidemiologic Studies Depression Scale*; GDI: *Geriatric Depression Inventory*; PQEEPH: *Psychological Questionnaire for Emergent Event of Public Health*; DASS: *Depression, Anxiety and Stress Scale*; HADS: *Hospital Anxiety and Depression Scale*; HAMD: *Hamilton Depression Rating Scale*

**Table S3 The differences between before and during the COVID-19 pandemic**

|  |  | **2017–2019 (pre-COVID-19)** | | **2020–2023 (during the pandemic)** | | **p-value** |
| --- | --- | --- | --- | --- | --- | --- |
| **Variables** | **Categories** | **Number of Studies** | **P (95%CI)** | **Number of studies** | **P (95%CI)** |  |
| Sex | Male | 31 | 21.0 (17.8–24.3) | 22 | 20.7 (14.8–26.7) | <0.001 |
|  | Female | 32 | 26.6 (22.2–30.9) | 22 | 25.9 (18.2–33.7) | <0.001 |
| Age group | 60-69 | 19 | 24.3 (18.4–30.2) | 7 | 25.6 (6.7–44.4) | <0.001 |
|  | 70-79 | 16 | 27.4 (20.5–34.3) | 6 | 32.0 (7.3–56.8) | <0.001 |
|  | ≥80 | 13 | 31.0 (21.8–40.3) | 8 | 33.7 (18.1–49.4) | <0.001 |
| Living area | Rural | 11 | 26.5 (15.5–37.4) | 8 | 26.4 (15.6–37.2) | <0.001 |
|  | Urban | 10 | 19.5 (14.6–24.4) | 6 | 20.0 (11.7–28.4) | <0.001 |
| Education level | Primary and below | 22 | 24.0 (19.3–28.7) | 14 | 31.0 (22.5–39.5) | <0.001 |
|  | Junior high school | 14 | 18.9 (14.2–23.6) | 9 | 23.5 (13.0–34.1) | <0.001 |
|  | Senior high school and above | 17 | 18.1 (14.6–21.5) | 9 | 17.9 (8.5–27.3) | <0.001 |
| Marital status | Single/Divorce/Widowed | 21 | 29.8 (23.7–35.8) | 16 | 32.5 (23.0–42.0) | <0.001 |
|  | Married | 21 | 23.6 (19.0–28.2) | 16 | 22.0 (14.7–29.3) | <0.001 |
| Residential status | Live alone | 17 | 29.7 (23.0–36.4) | 8 | 36.0 (20.0–52.1) | <0.001 |
|  | Not live alone | 17 | 23.0 (18.9–27.0) | 8 | 17.7 (10.7–24.6) | <0.001 |

**Table S4 Sensitivity analysis results for studies from 2017 to 2019**

| **Deleted Study** | **Effect (%)** | **95% CI** |
| --- | --- | --- |
| Sun et al., 2020 | 24.9 | 21.2–28.6 |
| Peng et al., 2022 | 25.0 | 20.9–29.1 |
| Hu et al., 2022 | 25.1 | 21.0–29.3 |
| Zhao et al., 2022 | 25.2 | 21.0–29.3 |
| Wang et al., 2021 | 25.3 | 21.2–29.5 |
| Rong et al., 2021 | 25.3 | 21.2–29.3 |
| Li et al., 2023 | 25.3 | 21.4–29.1 |
| Li et al., 2021 | 25.4 | 21.3–29.6 |
| Gao et al., 2022 | 25.4 | 21.3–29.5 |
| Hou et al., 2022 | 25.5 | 21.4–29.7 |
| You et al., 2023 | 25.5 | 21.3–29.6 |
| Xiong et al., 2023 | 25.5 | 21.4–29.7 |
| Li et al., 2021 | 25.6 | 21.4–29.7 |
| Yang et al., 2021 | 25.6 | 21.5–29.8 |
| Li et al., 2022 | 25.6 | 21.4–29.7 |
| Zhang et al., 2019 | 25.7 | 21.5–29.9 |
| Zhang et al., 2022 | 25.7 | 21.5–29.8 |
| Chang et al., 2023 | 25.7 | 21.5–29.9 |
| Wang et al., 2024 | 25.7 | 21.5–29.9 |
| Chen et al., 2019 | 25.8 | 21.7–30.0 |
| Ma et al., 2020 | 25.8 | 21.6–30.0 |
| Ding et al., 2022 | 25.8 | 21.7–30.0 |
| Lin et al., 2020 | 25.8 | 21.6–29.9 |
| Dai et al., 2019 | 25.8 | 21.6–30.0 |
| Xiong et al., 2022 | 25.8 | 21.7–30.0 |
| Ding et al., 2018 | 25.9 | 21.7–30.1 |
| Gu et al., 2020 | 25.9 | 21.8–30.1 |
| Zhao et al., 2020 | 25.9 | 21.7–30.1 |
| Guo et al., 2022 | 25.9 | 21.7–30.0 |
| Wang et al., 2022 | 26.0 | 21.7–30.4 |
| Han et al., 2021 | 26.0 | 21.8–30.2 |
| Li et al., 2022 | 26.1 | 21.9–30.3 |
| Yang et al., 2021 | 26.1 | 21.9–30.3 |
| Zhang et al., 2021 | 26.1 | 21.9–30.4 |
| Yan et al., 2022 | 26.1 | 21.9–30.3 |
| Jiang et al., 2022 | 26.1 | 21.9–30.3 |
| Lin et al., 2021 | 26.1 | 21.8–30.3 |
| Liang et al., 2021 | 26.1 | 21.9–30.3 |
| Liu et al., 2021 | 26.1 | 21.9–30.3 |
| Chu et al., 2020 | 26.1 | 21.9–30.3 |
| Yuan et al., 2023 | 26.1 | 21.8–30.4 |
| Wang et al., 2023 | 26.1 | 21.9–30.3 |
| Liu et al., 2021 | 26.2 | 21.8–30.5 |
| Zhai et al., 2023 | 26.2 | 21.9–30.4 |
| Wang et al., 2022 | 26.2 | 22.0–30.4 |
| Xu et al., 2022 | 26.2 | 22.1–30.4 |
| Jiang et al., 2022 | 26.2 | 22.0–30.4 |
| Wu et al., 2020 | 26.2 | 22.1–30.4 |
| Zeng et al., 2023 | 26.2 | 22.0–30.4 |
| Liu et al., 2022 | 26.3 | 22.1–30.4 |
| Qiu et al., 2020 | 26.8 | 21.8–30.3 |

**Table S5 Sensitivity analysis results for studies from 2020 to 2023**

| **Deleted Study** | **Effect (%)** | **95% CI** |
| --- | --- | --- |
| Mao et al., 2022 | 22.7 | 18.8–26.6 |
| Yang et al., 2024 | 23.2 | 19.3–27.2 |
| Li et al., 2024 | 23.2 | 19.2–27.1 |
| Guo et al., 2024 | 23.2 | 19.2–27.2 |
| Hao et al., 2023 | 23.3 | 19.3–27.3 |
| Li et al., 2022 | 23.3 | 19.3–27.2 |
| Wu et al., 2020 | 23.3 | 19.5–27.0 |
| Yu et al., 2022 | 23.4 | 19.5–27.4 |
| Liu et al., 2022 | 23.4 | 19.4–27.4 |
| Liu et al., 2022 | 23.4 | 19.4–27.5 |
| Zhang et al., 2024 | 23.4 | 19.6–27.2 |
| Lu et al., 2022 | 23.5 | 19.5–27.5 |
| Lu et al., 2023 | 23.5 | 19.5–27.5 |
| Huang et al., 2024 | 23.5 | 19.6–27.5 |
| Zhao et al., 2023 | 23.5 | 19.5–27.6 |
| Liang et al., 2021 | 23.6 | 19.6–27.6 |
| He et al., 2022 | 23.6 | 19.6–27.6 |
| Yang et al., 2023 | 23.6 | 19.6–27.7 |
| Ge et al., 2023 | 23.6 | 19.6–27.6 |
| Xu, 2022 | 23.7 | 19.7–27.7 |
| Liu et al., 2024 | 23.8 | 19.7–27.8 |
| Jiang et al., 2024 | 23.8 | 19.8–27.9 |
| Xiong et al, 2024 | 23.8 | 19.8–27.9 |
| Qin et al., 2022 | 23.9 | 19.9–28.0 |
| Cui et al., 2022 | 23.9 | 19.9–27.9 |
| Ding et al., 2022 | 23.9 | 19.9–27.9 |
| Wang et al., 2021 | 23.9 | 19.8–28.1 |
| Zhao et al., 2024 | 23.9 | 19.8–28.0 |
| Su et al., 2023 | 23.9 | 19.8–27.9 |
| Gan et al., 2024 | 23.9 | 19.9–28.0 |
| Li et al., 2022 | 24.0 | 19.9–28.0 |
| Li et al., 2022 | 24.0 | 20.0–28.1 |
| Hou et al., 2022 | 24.0 | 20.0–28.1 |
| Liu et al., 2021 | 24.0 | 19.9–28.0 |
| Zhou et al., 2021 | 24.0 | 19.9–28.0 |
| Wang et al., 2020 | 24.0 | 19.9–28.2 |
| Liu et al., 2024 | 24.0 | 19.9–28.0 |
| Lin et al., 2023 | 24.0 | 20.0–28.1 |
| Zhang et al., 2024 | 24.0 | 19.9–28.0 |
| Hu et al., 2023 | 24.0 | 19.9–28.0 |
| Peng et al., 2024 | 24.0 | 19.9–28.2 |
| Chen et al., 2023 | 24.1 | 20.0–28.2 |
| Qin et al., 2022 | 24.1 | 20.0–28.1 |
| Liu et al., 2022 | 24.1 | 20.0–28.2 |
| Wang et al., 2020 | 24.1 | 20.0–28.2 |
| Li et al., 2022 | 24.1 | 20.1–28.2 |
| Zong et al., 2022 | 24.1 | 20.0–28.3 |
| Liu et al., 2023 | 24.1 | 20.1–28.1 |
| Yu et al., 2024 | 24.2 | 20.0–28.5 |
| He et al., 2024 | 24.2 | 19.9–28.6 |

**Table S6 Sensitivity analysis results for studies from 2020 to 2022**

| **Deleted Study** | **Effect (%)** | **95% CI** |
| --- | --- | --- |
| Mao et al., 2022 | 22.0 | 18.1–25.8 |
| Li et al., 2024 | 22.5 | 18.6–26.3 |
| Guo et al., 2024 | 22.5 | 18.6–26.5 |
| Hao et al., 2023 | 22.6 | 18.7–26.5 |
| Li et al., 2022 | 22.6 | 18.7–26.5 |
| Wu et al., 2020 | 22.6 | 18.9–26.2 |
| Yu et al., 2022 | 22.7 | 18.8–26.7 |
| Liu et al., 2022 | 22.7 | 18.7–26.7 |
| Liu et al., 2022 | 22.7 | 18.8–26.7 |
| Zhang et al., 2024 | 22.7 | 19.0–26.4 |
| Lu et al., 2022 | 22.8 | 18.9–26.7 |
| Lu et al., 2023 | 22.8 | 18.9–26.7 |
| Liang et al., 2021 | 22.9 | 19.0–26.9 |
| Zhao et al., 2023 | 22.9 | 18.9–26.8 |
| Xu, 2022 | 23.0 | 19.0–27.0 |
| He et al., 2022 | 23.0 | 19.0–26.9 |
| Yang et al., 2023 | 23.0 | 19.0–26.9 |
| Ge et al., 2023 | 23.0 | 19.0–26.9 |
| Liu et al., 2024 | 23.1 | 19.1–27.1 |
| Cui et al., 2022 | 23.2 | 19.3–27.2 |
| Ding et al., 2022 | 23.2 | 19.3–27.2 |
| Jiang et al., 2024 | 23.2 | 19.2–27.1 |
| Xiong et al., 2024 | 23.2 | 19.2–27.1 |
| Qin et al., 2022 | 23.3 | 19.3–27.3 |
| Li et al., 2022 | 23.3 | 19.4–27.3 |
| Wang et al., 2021 | 23.3 | 19.2–27.3 |
| Liu et al., 2021 | 23.3 | 19.3–27.3 |
| Zhou et al., 2021 | 23.3 | 19.3–27.3 |
| Zhao et al., 2024 | 23.3 | 19.2–27.3 |
| Liu et al., 2024 | 23.3 | 19.3–27.3 |
| Zhang et al., 2024 | 23.3 | 19.3–27.3 |
| Hu et al., 2023 | 23.3 | 19.3–27.3 |
| Gan et al., 2024 | 23.3 | 19.3–27.2 |
| Chen et al., 2023 | 23.4 | 19.4–27.4 |
| Qin et al., 2022 | 23.4 | 19.4–27.4 |
| Li et al., 2022 | 23.4 | 19.4–27.4 |
| Hou et al., 2022 | 23.4 | 19.4–27.4 |
| Wang et al., 2020 | 23.4 | 19.4–27.4 |
| Wang et al., 2020 | 23.4 | 19.3–27.4 |
| Liu et al., 2023 | 23.4 | 19.5–27.4 |
| Lin et al., 2023 | 23.4 | 19.4–27.4 |
| Peng et al., 2024 | 23.4 | 19.3–27.5 |
| Liu et al., 2022 | 23.5 | 19.5–27.5 |
| Li et al., 2022 | 23.5 | 19.5–27.5 |
| Zong et al., 2022 | 23.5 | 19.4–27.6 |
| Yu et al., 2024 | 23.6 | 19.3–27.8 |
| He et al., 2024 | 23.6 | 19.2–27.9 |
