# Supplementary material for: Differences in depression prevalence among older adults in China before and during the COVID-19 pandemic: a systematic review and meta-analysis
Source: PeerJ. 2025 Apr 11;13:e19251. doi: 10.7717/peerj.19251 (PMC11995894; doi:10.7717/peerj.19251)
Supplement: Supplemental Information 3 [file peerj-13-19251-s003.docx]

**1. The rationale for conducting the systematic review/meta-analysis.**

The COVID-19 pandemic has profoundly impacted global mental health, particularly among elderly populations. Despite an extensive body of research on depression in older adults, the effects of different stages of the COVID-19 pandemic on depression prevalence among the elderly in China remain underexplored. Given China’s unique pandemic response strategies, such as the “dynamic zero-COVID-19” policy and strict mobility restrictions, a systematic review and meta-analysis is critical to understanding the nuanced changes in depression prevalence among this vulnerable population. This analysis aims to provide empirical insights into how these policies influenced mental health outcomes, identify high-risk subgroups, and offer data-driven recommendations for future public health interventions.

**2. The contribution to knowledge in light of previously published reports.**

Previous meta-analyses have primarily focused on generalized impacts of COVID-19 on mental health, often neglecting the elderly or lacking stratified analyses by pandemic stages. This study builds on prior research by systematically examining depression prevalence across different COVID-19 stages (2017–2019, January–April 2020, May–December 2020, and 2021–2024) and comparing pre-pandemic and pandemic periods in the Chinese elderly population. It contributes to the field by:

(1) Providing the most comprehensive analysis of depression prevalence in Chinese elderly individuals during COVID-19, integrating findings from 101 studies with 264,758 participants.

(2) Identifying demographic and social factors contributing to changes in depression prevalence, including urban-rural disparities, educational levels, marital status, and living arrangements.

(3) Conducting further statistical analyses based on the meta-analytic results to explore trends in depression prevalence across various subgroups and periods.

(4) Highlighting the efficacy of China’s public health interventions and their long-term psychological consequences on the elderly.

This systematic review and meta-analysis fills critical gaps in understanding depression trends during the COVID-19 pandemic, offering a valuable reference for policymakers and researchers addressing mental health challenges in similar crises.
